# Clustering- and statistic-based approach for detection and impact evaluation of faults in end-user substations of thermal energy systems

**DOI:** 10.1038/s41598-024-82103-5

**Published:** 2024-12-31

**Authors:** Samanta A. Weber, Michael Fischlschweiger, Dirk Volta, Ulf Rieck-Blankenburg

**Affiliations:** 1 Energy and Life Science, University of Applied Sciences Flensburg, 24943 Flensburg, Germany; 2 Chair of Technical Thermodynamics and Energy Efficient Material Treatment, Institute of Energy Process Engineering and Fuel Technology, Clausthal University of Technology, 38678 Clausthal-Zellerfeld, Germany; 3 Business Segment Networks, Stadtwerke Flensburg GmbH, 24939 Flensburg, Germany

**Keywords:** Heat network efficiency, K-Means clustering, Fault management, Thermal energy systems, Energy grids and networks, Energy grids and networks

## Abstract

In response to climate change mitigation efforts, improving the efficiency of heat networks is becoming increasingly important. An efficient operation of energy systems depends on faultless performance. Following the need for effective fault detection and elimination methods, this study suggests a three-step workflow for increasing automation in managing defective substations on the user level within heat networks. The work focuses on a model region in northern Germany. The local heat network provides data in roughly hourly intervals, including the supply and return temperatures and the volume flow of the substations. Firstly, this study identifies common indicators of faults using k-means clustering analysis of the temperature data and expert knowledge: an exceeded return temperature level, very low cooling, and inverted temperature readings. With these indicators, the subsequent statistical identification approach confirms the successful detection of affected substations, with common diagnoses including disabled return temperature limitation units, defective motoric valves, and faults in the storage control. Lastly, the study evaluates the impact of faults on the system efficiency. Combining the temperature and the volume flow data, the workflow quantifies the negative influence of a fault, enabling the prioritization of fault elimination measures in practical application to enhance the overall system efficiency.

**Supplementary Information:**

The online version contains supplementary material available at 10.1038/s41598-024-82103-5.

## Introduction

Heating systems are widely considered the most suitable solution for areas of high heat demand density^1–3^. However, transitioning existing systems to the 4th generation of district heating, which operates at lower temperatures, poses significant challenges^4^. Effective methodologies are required to reduce operating temperatures while accounting for existing network infrastructure.

Substations, which transfer heat from the network to users, are critical components regarding the heat network efficiency and often exhibit faults^5–8^. Faults in the substations typically require increasing either the supply temperature or the volume flow^9^through the substation, counteracting efforts to reduce system temperatures. Fault detection at the user level is crucial for enhancing thermal energy systems’ efficiency, as single users can significantly impact the overall network performance^10^. Effective fault management could achieve half of the required temperature reduction for the transformation to 4th generation systems^8^. Besides improving efficiency, fault detection enhances reliability and safety^11^and can achieve cost savings^12^. Thus, developing feasible fault detection methods for user-level substations is essential.

Fault detection methods are mostly data-driven as they typically require large quantities of measurement data^13^. These include supervised (regression, classification) and unsupervised (e.g., clustering) models^12^. The standard strategy of fault detection methods is to compare the measured state of the system with an expected, faultless state^13^. With the rise of smart meters, these approaches have become increasingly relevant^14^, yet existing methods face challenges like limited scalability, significant manual intervention, and complexity^5,9,14,15^, counteracting the practical application.

The literature emphasizes the need for effective fault detection^5,8,9^. For instance, Månsson et al.^9^ underline the relevance of a well-operating model for fault detection, while Buffa et al^5^. highlight the advantages of machine learning despite its high data requirements. Manual fault detection is time-intensive^5^, and substations with low overall heat demand are often assigned lower priority or not monitored at all^9^. Yet, aiming for lower supply temperatures, this currently existing fault tolerance^8^ should not be accepted but regarded as an optimization potential. In a related context, Fabre et al.^17^focus on improving user heating systems by reducing return temperatures. Given the potentially large number of substations, Ref^17^. emphasizes the importance of prioritizing those with the most significant negative impact. For this, a limited number of operators consider the heat or volume flow of the station^7^. Thus, fault detection methods should be cost- and data-effective enough to consider all substations in a system rather than only those with the highest heat demand and quantify the negative impact of the fault on the heating system.

Further studies emphasize the need for reliable, scalable, cost-efficient, and simple solutions that minimize manual processing^14,15^. Data accessibility is one primary inhibitor, e.g., for secondary-side fault detection^16^. The objective must be to use easily accessible data (e.g., primary-side data, not secondary-side data, often challenging to collect) to simplify fault diagnosis and minimize manual intervention^15^.

Automation is essential for effective fault management but requires increased efforts in data labeling^12,19^. A majority of studies focus on anomaly detection with unsupervised learning because labeled data is scarce^12^. As faults are a subgroup of anomalies, existing approaches can lead to an overextension of the resources an operator can invest^5,12^. Ref^12^. concludes that an effort should be put into further advancing actual fault detection alongside anomaly identification.

Clustering is a commonly applied method for grouping data and, thus, generating labels. Calikus et al.^10^ and Gianniou et al.^18^introduce clustering methods to automatically identify user data patterns in heat networks to increase knowledge of thermal energy systems. Ref^10^. emphasizes the relevance of continuous monitoring for detecting errors. The approach allows experts to outline substations that do not meet the behavior expected for the assigned control strategy^10^ and, thus, aids in labeling data.

However, there is no standard monitoring approach to fault detection on the substation level^7^. Ref^7^. identifies monitoring the return temperature, the temperature difference between the supply and return temperatures, and consumption or over-consumption (measured supply in comparison to an expected value) as existing approaches for fault detection. Another existing label for fault detection is the loss of user comfort^21,22^. Certain studies define a fault as always affecting user comfort and assign the term anomalies to the remaining phenomena^21,22^. Others, e.g., Ref^7,17,20^., express that inhabitants often do not notice faults in substations if no loss of comfort occurs. However, independent of the chosen terminology, the impact on user comfort can be insufficient as a criterion for fault detection. Therefore, methods for fault detection should be more standardized.

Certain studies showcase the application of the time-series data collected for the temperatures and the volume flow for fault detection. In the practical application of fault diagnosis, time dependencies, e.g., strongly relying on the information in the sequential order of time-series data, inflict an additional dimension and, hence, increase the complexity of the approach. In the context of reducing time dependency, Gadd et al.^6^ suggest the “excess flow” (increased volume through substation caused by a fault) and the “thermal signature” (correlation of the temperature difference achieved by the substation and the outside temperature) as indicators of substation performance. In a subsequent study, Gadd et al.^8^ inquire about the nature of occurring faults in user substations, found to be unsuitable heat load patterns, a low average annual temperature difference, and poor substation control. Calikus et al.^20^establish a method for fault detection working with the so-called “heat power signature” (or “energy signature”, the correlation of heat demand and outside temperature^6,8,15,20,23^) for the detection of errors in heat networks. The studies^6,8,15,20,23^ suggest that relevant information for fault detection can be obtained with limited consideration of the time dimension. Thus, research should focus on investigating methods that reduce the dependence on the sequential order of time-series data and develop strategies to simplify data pre-processing without losing relevant temporal information.

An additional benefit of fault management is increasing measurement data quality for research purposes. Ref^24–27^. highlight the importance of high-quality data for advancing heat network models. Improving fault detection directly increases network efficiency. However, it also enhances data quality and, thus, indirectly supports more advanced data-driven system modeling, which is considered highly relevant for further improving system efficiency^28^.

This study aims to develop an effective workflow for using substation data for fault detection on the user level in heating systems, with a focus on achieving a high degree of automation. The objectives of effectiveness and a high degree of automation shall be assessed by the following:

First, the workflow should reduce manual intervention. This study aims to contribute to this objective by utilizing available primary-side system data and encoding the employed data pre-preparation methods for automation. Also, the number of substations to be manually assessed shall be reduced, and the detection of faulty substations shall be fully automated.

Second, the method should lessen the reliance on the sequential order of time-series data, ensuring a more flexible and efficient process. Therefore, fault detection shall occur based on the sample distribution rather than by exploiting the information that the sequential order of the data can provide.

Third, all substations, regardless of their respective size, shall be monitored. To achieve this, the faults’ impact on the heating network shall be quantified, allowing for the prioritization of elimination measures. The impact quantification shall also be fully automated.

The article is structured as follows: Section "Materials and methods" outlines the research methodology. The study introduces the framework of the fault identification methodology, presents the model region, and provides the measurement architecture for data collection. Additionally, this work demonstrates the concept of the temperature signature of substations and introduces the necessary steps for data pre-preparation to use the k-means clustering approach. Further, the statistical identification process, as well as the quantification of the faults’ impact, are explained. Section "Results" presents the results of the case study, including the identification and labeling of indicators with clustering and expert knowledge, fault detection with statistical methods, and the impact quantification of the faults. Furthermore, this work presents certain special cases of detected temperature signatures. Section "Discussion" provides the discussion. Section "Conclusions" finishes with the conclusions.

## Materials and methods

### Fault detection framework

**Fig. 1:**
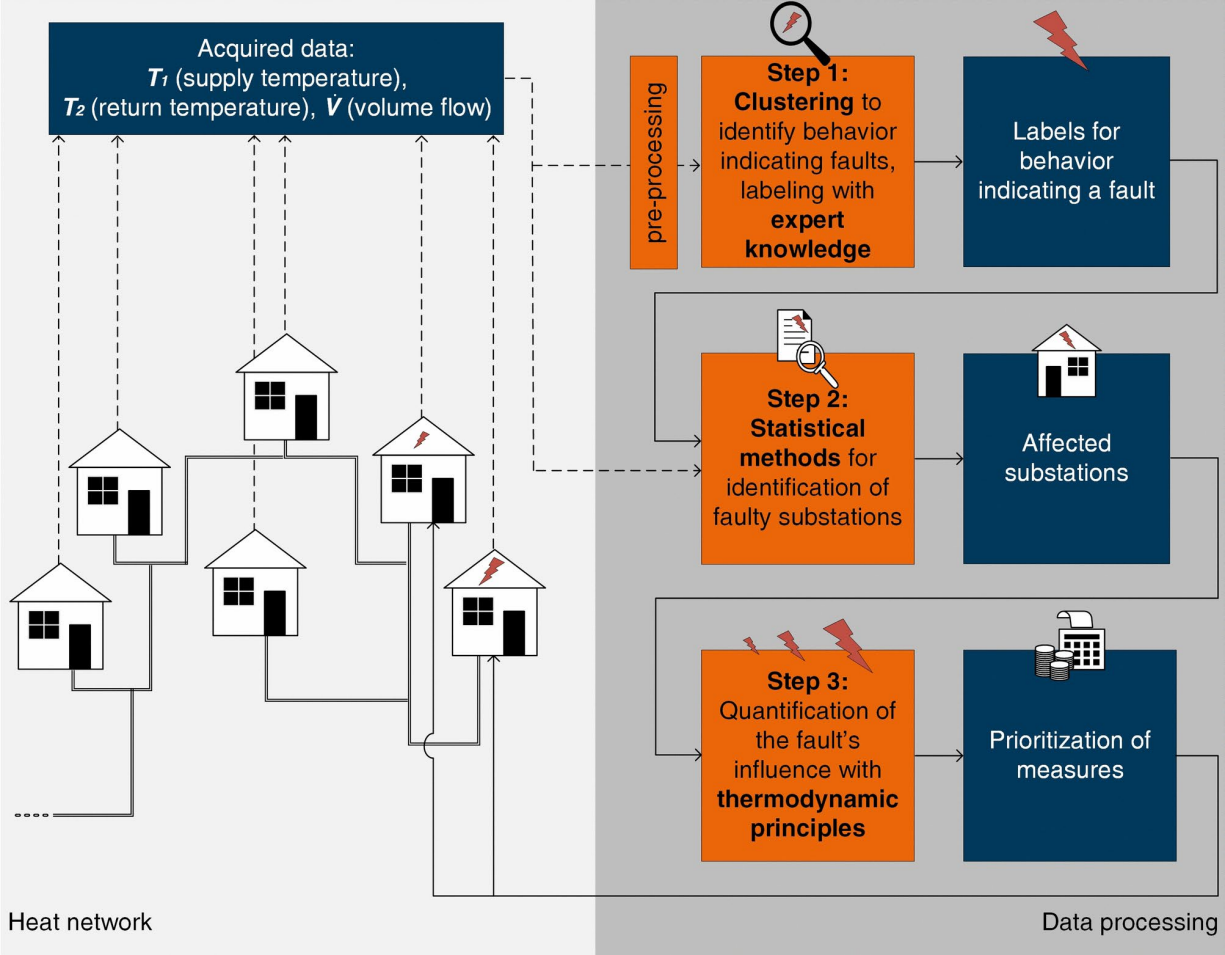
Fault identification workflow. The fault identification occurs in three steps, namely identification of the relevant faults with clustering, providing groups of faults to be labeled with expert knowledge, subsequently establishing statistical features to automatically detect substations affected by the labeled faults and assign a numerical value to quantify the (negative) impact of the faulty substation on the system to prioritize measures with thermodynamic fundamental relationships.

In this work, fault detection occurs in a three-step workflow, highlighted in Fig. 1. First, typical patterns of substation data indicating faults are determined and labeled with clustering and expert knowledge. Second, a statistical approach for detecting faulty substations, automated by encoding, is developed. Third, the same code assigns a numerical value to quantify the fault’s impact on the overall system.

Figure 1 illustrates the processing steps in orange and the obtained information in dark blue. The applied methods are highlighted in bold. As visible, the acquired raw data of the substations in the model region is pre-prepared prior to subsequent processing. As this study focuses on the interdependencies of the user substations and the heating system, we use the terms “fault” and “error” to describe substation behavior that has a disadvantageous impact on the heat network efficiency.

The pre-prepared data is then input into the workflow. For the first step, this study uses k-means clustering to establish the common (faultless) pattern as the reference for identifying anomalous observations in the temperature data of the substations in the model region. To achieve transferability, this work takes advantage of the statistical nature of k-means rather than explicitly defining a specific faultless reference pattern. Clustering-based methods are unsupervised and can identify patterns among data and assign subsets with similar patterns to groups. This step might require comparatively high computational resources. As the clustering provides no reason for the decision, expert knowledge is required to provide labels for the groups. It is used to label statistically accessible indicators to detect defective substations. In this study, patterns indicating faults are labeled with statistically accessible features.

In the second step, these indicators can be used to identify faulty substations automatically through an encoded statistical process. The acquired labels and the raw data are the inputs. Such a statistical evaluation typically requires less computational effort than clustering. However, this study’s method demands a feature indicative of an error, which the clustering approach provides. The statistical approach returns the substations affected by at least one of the faults labeled in the first step.

Thirdly, information on the intensity of the negative impact of the detected faulty behavior on the heating system can be provided, relying on the thermodynamic relationships of volume flow and temperatures. The information on the occurrence of a fault obtained in the second step is insufficient, as it does not provide any measure of prioritizing elimination actions. This can overstrain the operator’s resources for fault management. Hence, the code automatically returns a numeric quantification of the fault’s impact on the system.

Automation enables repetitive conduction of fault detection and impact evaluation. Therefore, conducting the second and third steps of the suggested workflow at regular intervals using the recently recorded data outlines existing, newly occurring, and reoccurring faults.

### Model region and data acquisition

**Fig. 2:**
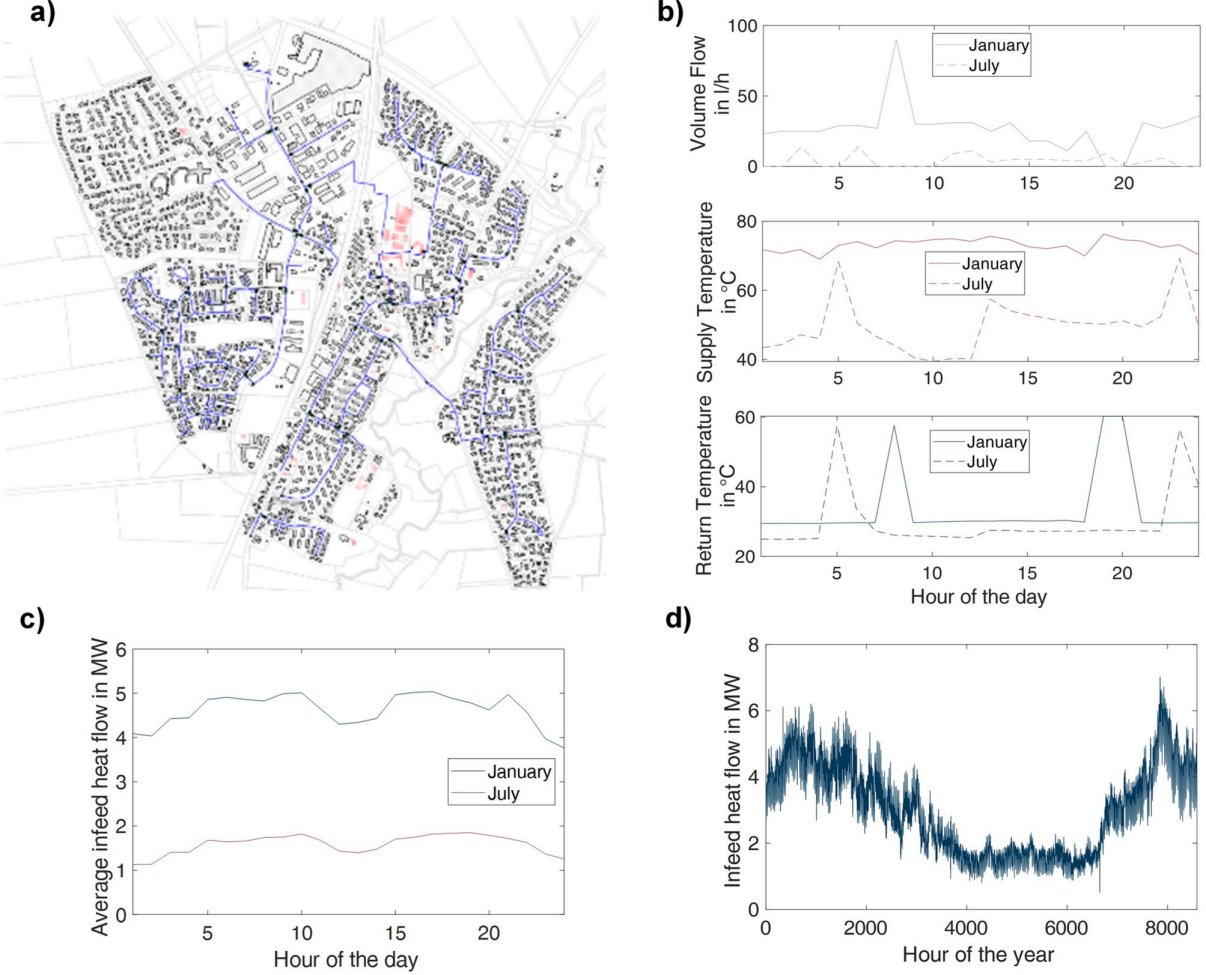
Network structure of the model region, data of one substation and for the infeed heat flow for a day in January and July, and dynamic data of the infeed heat flow over one year. The model region in **a**) is a centrally supplied district heating network with predominantly stub lines and one closed circle line; the data in **b**) visualizes the dynamics of one substation over a day; **c**) highlights that the infeed heat flow over a day follows two demand peaks and the general level depends on the season, further visualized by **d**) the heat infeed over the year.

This study employs exemplary data of the supply and return temperature as well as the volume flow recorded in roughly hourly time steps between 1^st^ January and 31^st^ December 2023, of a centrally supplied district heating system in Tarp, northern Germany, with 486 substations. Figure 2 a) displays the general structure of the system, Fig. 2 b) shows the recorded values of the volume flow, the supply, and the return temperature for a day of January (mid-heating season) and July (mid-summer) for one exemplary building in the heating system. Figure 2 c) depicts the infeed heat flow for the same days and Fig. 2 d) the infeed heat flow over the hour of the year.

For the infeed of the heat network, the supply temperature ranges between 70 and 105 °C. The substations are designed to receive the minimum supply temperature of 70 °C for an outside temperature ≥ 15 °C and up to 100 °C for an outside temperature ≤−10 °C. Currently, the return temperature received at the infeed plant reaches values between 50 and 55 °C, which is a comparatively high value compared to more modern heating systems.

The heat network mainly covers the heat demand of individual and multi-family homes. Certain buildings share a common substation. One substation supplies a living quarter (see the upper left corner of Fig. 2 a). One industrial customer and buildings used by the public (e.g., schools, gyms, churches) are connected to the system as well.

**Fig. 3:**
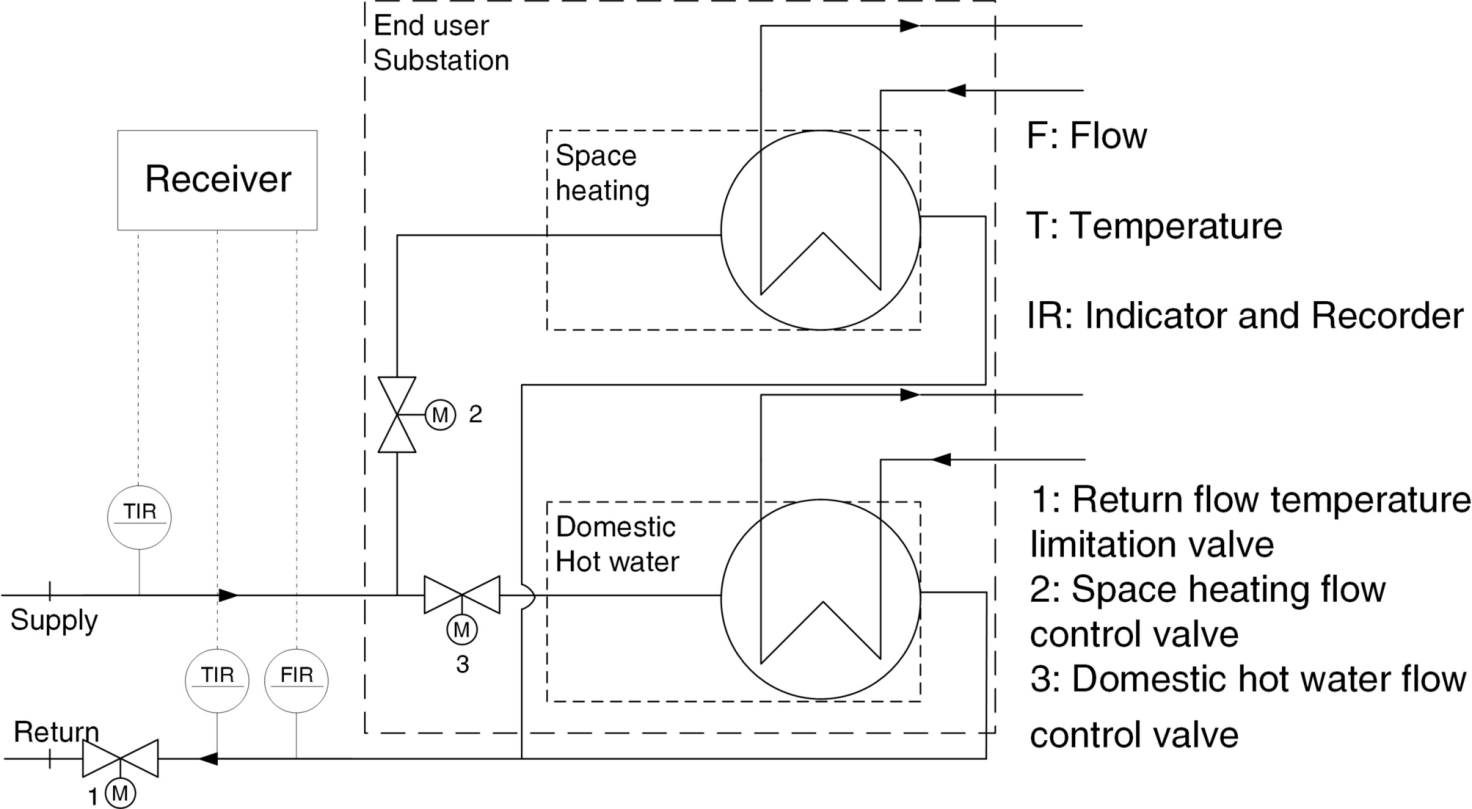
Exemplary measurement architecture of a substation on the user level in the model region in flow diagram. The heat supply can cover both domestic hot water and space heating or the latter only and is measured as the combined demand on the primary side of the heat exchanger with two Temperature Indicators and Recorders (TIR) and a device for the volume flow measurement (FIR).

The fault management method of this study rests predominantly on the data of the individual substations, as captured exemplarily in Fig. 2 b). Users in the model region can receive heat for domestic hot water and space heating or the latter only. Further, it is possible to heat the domestic hot water directly through the heat exchanger or use a storage facility, which impacts the temperature level required for the supply and the resulting return temperature. Figure 3 depicts a typical measurement architecture on the end-user level within the model region.

The measurement architecture captures the sum of the heat supplied for domestic hot water and space heating. The motoric valves, as actors, determine the volume flow values. As the scheme in Fig. 3 is an advantageous setup, it should be mentioned that the real-case scenarios can (strongly) differ, possibly influencing the efficiency. For the direct domestic hot water supply without a storage facility, a low flow rate of primary side heat carrier is required for times of no demand. This maintains the level of temperature needed when demand occurs again. Hence, in this specific case, no complete stagnation of the substation can occur, but the volume flow is limited to avoid excessively high return temperatures.

If measures for eliminating the faults lie in the user’s sphere, the operator can only give advice. Ref^7^., therefore, suggests that a good relationship with the user is highly relevant in fault elimination. However, legally binding contracts containing threshold values to be adhered to can exist, as in the model region. Specifically, the return temperature maximum to be adhered to on the average is 55 °C. An excessive return temperature causes relevant heat losses in the return line. Also, it is indicative of a low cooling of the heat carrier, which occurs for values of the volume flow surpassing the optimum. Thus, the hydraulic load of the system is affected. Additionally, the losses in the supply line are increased: for a higher capacity flow (product of volume flow and heat capacity), the mean overtemperature of the supply line rises. Therefore, a user’s substation with a high return temperature has a substantial negative impact on the system. The operator determined that the boundary of 55 °C is the maximum level to limit the network losses to an acceptable level. As the user substations are the property of the user and also the user’s responsibility, the restriction to 55 °C further aims to provide a legal foundation for the operator to oblige the user to take measures.

It should be acknowledged that the limit value of 55 °C is specific to the model region and can appear high compared to other heating systems. The value can be adjusted, e.g., for more modern and modernized systems, as well as systems conceptualized under a different legal framework.

### Temperature signature of substations

In this study, following the terminology of Ref^20^., the term “temperature signature” refers to the supply temperature measurements of a substation displayed over the return temperature readings. Similarly to, e.g., Ref^6,8,20,23^., this study does not primarily rely on the information in the sequential order when treating the time-series data. Instead, the data are investigated with regard to the sample distribution. As temperatures alone indicate the efficiency of a thermal energy system^9^, this work hypothesizes that information on the occurrence of faults can be captured without relation to time, employing the supply and return temperature data of user substations for the identification of faults. Working with limited time dependency serves the objective of avoiding time-intensive pre-processing and reduces computational costs.

**Fig. 4:**
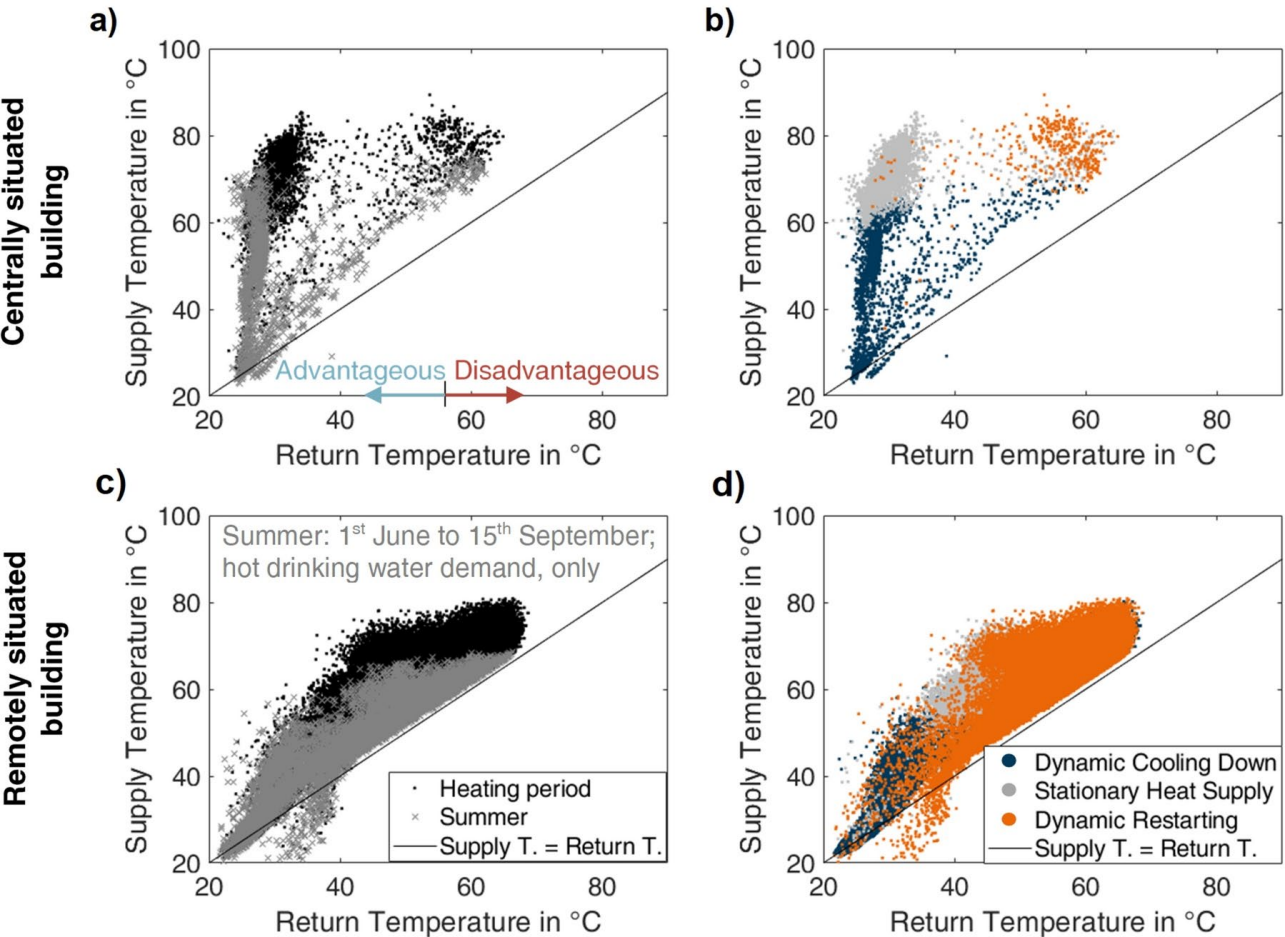
Temperature signatures of end-user substations outlining the line of equality for supply and return temperature. The substations in **a**), **b**) are centrally and in **c**), **d**) remotely positioned, and the diagrams enhance a), c) summer (domestic hot water demand, only; 1st June to 15th Sept., grey crosses) and heating period (space heating and domestic hot water demand, black dots), and b), d) the general operational modes existing for faultless behavior distinguished by the value of the volume flow (blue: zero for stagnation, grey: intermediate for stationary supply, orange: high for restarting), advantageous return temperatures defined as below 55 °C; the black solid line indicates equality of temperatures.

To further explain the concept, Fig. 4 contains temperature signatures for two substations. On the left, the plots distinguish between the summer (1^st^ June to 15^th^ September 2023) and the heating period, and on the right, between the existing operational modes. This study considers return temperatures above 55 °C as disadvantageous for the system efficiency. On the contrary, the lower the return temperature drops below 55 °C, the stronger the advantageous influence becomes. A black solid line in Fig. 4 indicates the equality of supply and return temperature.

The distance in pipe length from the infeed strongly influences the temperature signature of substations. Aiming for an efficient heat supply, the most advantageous user is situated close to the heat and power plant^29^ because the pipe length is minimal, and consequently, the heat losses are low. For this user, the supply temperature will range amongst the highest in the system. Such a situation is depicted in Fig. 4 a), b) (same substation as in Fig. 2 b)). On the other hand, if the user is remotely located (Fig. 4 c), d)), the heat carrier cools down in the distribution pipes and the connection pipe, which affects the temperature signature but does not necessarily indicate a fault.

Figure 4 a) and c) distinguish the summer (periods of usually only domestic hot water demand) and the heating season (periods of both space heating and domestic hot water demand) to visualize the seasonal impact. To account for seasonality, this study assumes that the data should be collected over at least one year, as a fault detection method should cover both periods. As the heat exchange for domestic hot water and space heating employs separate heat exchangers, faults can be categorized as affecting both heat exchangers or only one of the two by the distinction of the periods. Additionally, the point of time the fault occurred can be narrowed down. This kind of visualization shall, therefore, be applied in the following.

Additionally, the temperature signatures of the substations (Fig. 4, b), d)) show three general operational modes, distinguishable by the volume flow value.

- If the substation’s architecture allows for stagnation (zero volume flow, not possible for direct domestic hot water heating), the facility cools down (Fig. 4 b), d), blue dots) for periods of no demand, which is advantageous for the system. Stagnation occurs more often in summer (Fig. 4, grey crosses, a), c)), when domestic hot water is required only.
- In comparison, for periods of relatively constant heat demand, a state of approximately stationary heat supply will be reached (Fig. 4, grey dots, b), d)) with intermediate values of the volume flow. The substation’s control should adjust the volume flow to achieve the highest possible cooling of the heat carrier. This reduces the required volume flow and the resulting return temperature.
- When restarting the facility, the volume flow strongly increases, resulting in disadvantageously low temperature differences (Fig. 4, orange dots, b), d)). The low temperature difference places the values associated with high volume flow values on the right in the plots in Fig. 4. Hence, for an efficient substation, a limited number of samples should fall into this mode.

For additional, comprehensive visualization of the interdependency of volume flow and temperature signature, Fig. S1 and Fig. S2 (Supplementary Information) display the data in Fig. 4 with the extra dimension of the volume flow.

Measurements below the black solid line form a particular case caused by storage capacities in the system, and there are two possible explanations. Both rely on the fact that the return temperature measuring probe is often located physically closer to the heat exchanger:

- For a stagnating substation cooling down as in Fig. 4 a), b) (blue dots), the heat stored in the heat exchanger warms the return temperature measuring probe, while the supply temperature measuring probe (located further away) is unaffected.
- For a restarting facility like in Fig. 4 c), d) (orange dots), the cooled-down heat carrier in the distribution pipe must be replaced. A long stub lane connecting the substation to the system leads to a higher amount of water to be replaced. Until the hot heat carrier is received again, the heat carrier on the primary side can be warmed up by the secondary side heat carrier from the heat storage facility.

As can be seen, the operational mode influences the position of data points in the temperature signature. The time dependencies are related to the number of samples. Both combined lead to an accumulation of data points in the temperature signature, which is, therefore, relevant information that shall be used for clustering. To establish labels for faulty behavior, the faultless patterns must be known, which can range between the two extremes of locations depicted in Fig. 4.

### Clustering and expert knowledge for identification and labeling of indicators for faulty behavior

Clustering is an unsupervised machine learning technique^10^that aims to identify subgroups of data that differ from each other as strongly as possible but contain subsets of data that are as similar as possible^30^. This study employs the principle of k-means clustering. It splits a dataset of *i* observations into *k*clusters using two conditions^30^:

- The centroid of every cluster is the mean value of the observations in the cluster.
- Every observation is assigned to the cluster with the nearest centroid.

The algorithm itself contains five steps^30^:

1. k observations are chosen randomly as initial centroids from the dataset.
2. The distance of the remaining observations to the initial centroids is calculated.
3. Every remaining observation is assigned to the cluster with the nearest centroid.
4. The mean value of the newly established cluster is treated as new centroid.
5. Steps 2 to 4 are repeated until the algorithm converges.

The substations’ datasets form separate observations. As given, one observation is automatically chosen as the initial centroid for each cluster. Thus, applying the method more than once leads to differing results. This study takes advantage of this characteristic. Further, an ML method is unbiased and objective. This ensures that no subjective considerations of a (human) expert influence the results.

Data pre-preparation, i.e., feature engineering, must occur to enable comparability of the different substations’ datasets. Not all observations show equal numbers of measurements, so the number of data points in the observations must be equalized. Further, as described, the data accumulation in the temperature signatures fuses the information on the temperature, the volume flow, and the time dependencies and, hence, forms a relevant feature for evaluating a substation’s efficiency.

Therefore, to achieve comparability and integrate all the information, this study employs the representation of the temperature signatures as heat maps, which visualize data accumulation. For the processing steps in Fig. S3 (Supplementary Information), the scatter plots of the substations, as in Fig. 4, are regarded as grids with steps of 1 K in the two temperature directions. Evaluating the share of the overall number of samples falling into a specific grid position provides information for clustering.

Fig. S3 a) (Supplementary Information) shows the steps to obtain a heat map that represents data accumulation from the temperature signature. The modeling environment in this study is Matlab. As the predefined k-means clustering algorithm^31^ demands one-dimensional datasets, the two-dimensional datasets are parallelly transformed into single-row vectors by concatenating the rows of the grid from the lowest to the highest supply temperature, as visualized and described by the flow chart in Fig. S3 b) (Supplementary Information).

This step enables the comparison of observations with differing data collection rates as long as the recordings capture the relevant information on data accumulation. In contrast, limited numbers of collected samples, in particular, result in accumulations at specific points. These observations should be excluded. As a comparatively large number of substations achieves 90% of data collection, this study employs this share of 167 sets of end-user data for the analysis. For data privacy reasons, no traceable indicator identifies the individual substations.

The subsequent step to data pre-preparation is setting the clustering approach’s hyperparameters, in this case, the target number of clusters. As shown in Fig. 4, two general, expectable (faultless) behavioral patterns of substations exist: the advantageous and the rather disadvantageous patterns with regard to their influence on the system. The expectation is that these will form two clusters with a large number of assignments based on the expected (faultless) behavior.

Therefore, the hypothesis is that if the target number of clusters is set to three, this third cluster should contain only a small subset of the overall number of observations, most likely patterns similar to each other but differing strongly from those of the first two clusters. This study hypothesizes that the faulty and anomalous substation patterns will be found in this third, smaller cluster.

To identify all of these patterns present in the dataset, all substations prone to being assigned to the third cluster must be determined. This study takes advantage of the fact that the initial centroids of the clusters are chosen statistically by k-means. When the clustering is conducted repetitively for a sufficient number of times, all anomalous patterns will eventually form the third cluster at some point in the repetition.

Furthermore, this study relies on the circumstance that differing patterns should appear only for a small number of observations. Therefore, if the third cluster contains a high number of substations, the anomalous patterns are not successfully identified. With iterative manual testing, a suitable maximum number of assignments for a “small” cluster for the specific case of the model region’s data is determined to be five.

Apparently, certain anomalous patterns occur for a group of substations. If one of these forms the centroid of the third cluster, all similar patterns are assigned to this group. Gradually increasing the number defined as “small” from one to four leads to the detection of a small number of substations with indeed anomalous but unique patterns. For the number of five, considerably more substations with anomalous patterns are detected. Increasing the number of clusters further, a growing number of substations with only slightly anomalous patterns form clusters.

**Fig. 5:**
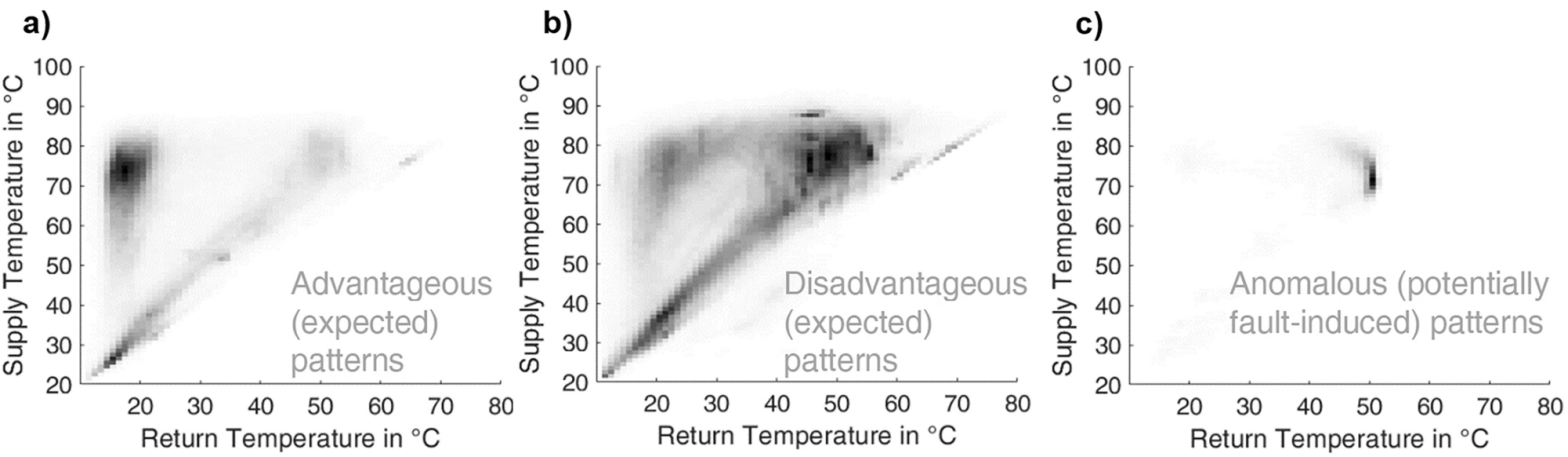
Heat maps of exemplary centroids of the clustering process with 3 clusters. Clusters **a**) advantageously operating substations and **b**) more disadvantageously operating substations contain the general behavior, while c) deviating operational patterns indicate anomalies.

Figure 5 displays the centroids of the resulting clusters for an exemplary execution of the process of splitting the data into three groups. To display the centroids, the single-row vectors returned by the k-means algorithm are reordered to the initial matrix shape of the 1-K-grid. Displaying the matrix as a heat map, lighter colors in Fig. 5 indicate lower ratios of the collected data falling into the grid position. Hence, the darker the color in a specific grid position, the more data were recorded in this grid position for the assigned substations.

Visibly, the first cluster contains the more advantageously operating substations (tendency to left in heat map of temperature signature), the second the more disadvantageous (tendency to right in heat map of temperature signature), and the third a share of substations with anomalous patterns, as expected. Similar results are returned for repetitive conduction, where the substations assigned to the third cluster change. This study, therefore, concludes that splitting the observations into three clusters serves the objective of outlining the differing temperature signatures but requires repetitive execution to detect all existing anomalies.

**Fig. 6:**
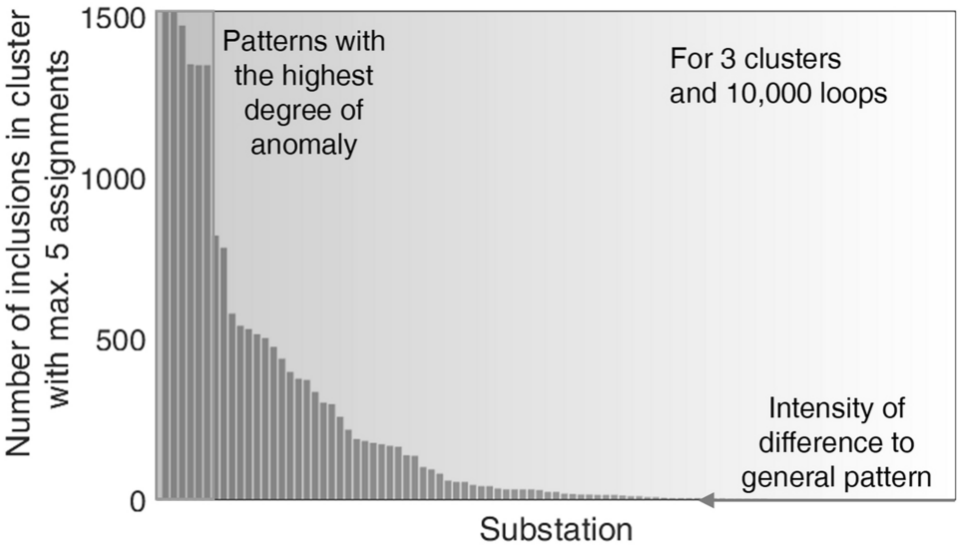
Number of inclusions of a substation into a small cluster. The number is indicative of the degree of deviation of the related temperature signature from the general pattern, with small cluster meaning max. 5 assignments; results for 10,000 executions of k-means clustering with a target value of 3 clusters.

To ensure all anomalous patterns are identified, this work executes the clustering process 10,000 times. For each substation, we calculate the number of times it is found in a cluster with a maximum of five assignments. The choice of 10,000 executions relies on the circumstance that the probability of the substations being in a small cluster becomes relatively stable when statistical effects level out. Further increasing the number of executions has a neglectable impact on the results. The number of times a substation is assigned to a small cluster indicates the degree to which the pattern differs from all others (“degree of anomaly”^20^). Figure 6 shows a bar graph for each substation with at least one assignment to a small cluster in descending order. The substations with the patterns with the highest degree of anomaly are found on the left.

The clustering assigns 95 of the 167 substations to a small cluster at least once. Therefore, clustering enables the objective reduction of the number of patterns that need to be manually investigated. The patterns that differ the strongest from the remaining dataset are assigned to a small cluster in up to 15% of the executions. It should be considered, as underlined in Ref^17^., that anomalous behavior is not directly an indication of a fault and that, if indeed a fault exists, the specific cause must be determined. Hence, for this study, all 95 of the substations’ patterns identified as anomalous were manually investigated with expert knowledge to obtain labels for the most relevant existing patterns indicative of the occurrence of a fault.

### Statistical identification of faulty substations and influence quantification

Statistical analysis is a method conducted traditionally^7^, as it is a computationally efficient option for fault detection. Additionally, as a physical representation of the indicator exists, the method is comprehensive. However, statistically accessible labels are required. Hence, it can be conducted only once labels for fault indication are established.

In this study, the application of the statistical fault identification thus depends on the labels acquired with the clustering approach and can only be conducted subsequently. Unlike the clustering approach, the statistical evaluation shows low sensitivity to the rate of collected data. Thus, with this second step of the statistical fault detection process, all 486 substations in the heating system can be covered.

Further, for an operator with limited resources, more than detecting the faults is required. The greater a fault’s influence on the system, the more critical eliminating the cause becomes. Historically, this has led to monitoring the substations supplying the most heat, possibly leaving faults in substations with low demand undetected^9^. Thus, all substations should be monitored to improve the system’s general operation. Evaluating the negative impact of the faulty substations on the system increases the benefit because the operator can prioritize fault elimination measures starting with the most disadvantageous substations.

Thus, the fault’s impact on the system should be quantified. This study employs the volume flow data recorded parallelly to the temperature data to reach this objective. The higher the volume flow, the stronger the impact on the system becomes.

Fig. S4 (Supplementary Information) provides a flow chart of the processing steps for the statistical fault detection and impact quantification. First, the indicators for the occurrence of a fault established in the first step of this three-step method are tested for each substation dataset individually. Second, it can be required to assess whether additional boundary conditions occur, which can be individual for the indicators (e.g., enough data collected, the general temperature level, etc.). Third, the heat transferred for the occurrence of the indicator is calculated as a quantification of the faults’ impact. Additionally, it is suggested that a substation is only marked as faulty if the calculated heat exceeds a threshold specific to the indicator and the model region. This maintains relevance and further narrows the meaningful information to the faults with the highest impact. The code automatically returns the relevant information and sorts the substations detected as faulty by the calculated heat values in descending order. Once the indicators are provided by step 1 of this study’s three-step method, the general framework can be explicitly encoded for steps 2 and 3.

## Results

### Clustering and expert knowledge-based identification of indicators for common faults in user substations

In this first step, this study aims to identify the most common behavioral patterns indicative of faults in the context of labeling data with the clustering algorithm.

**Fig. 7:**
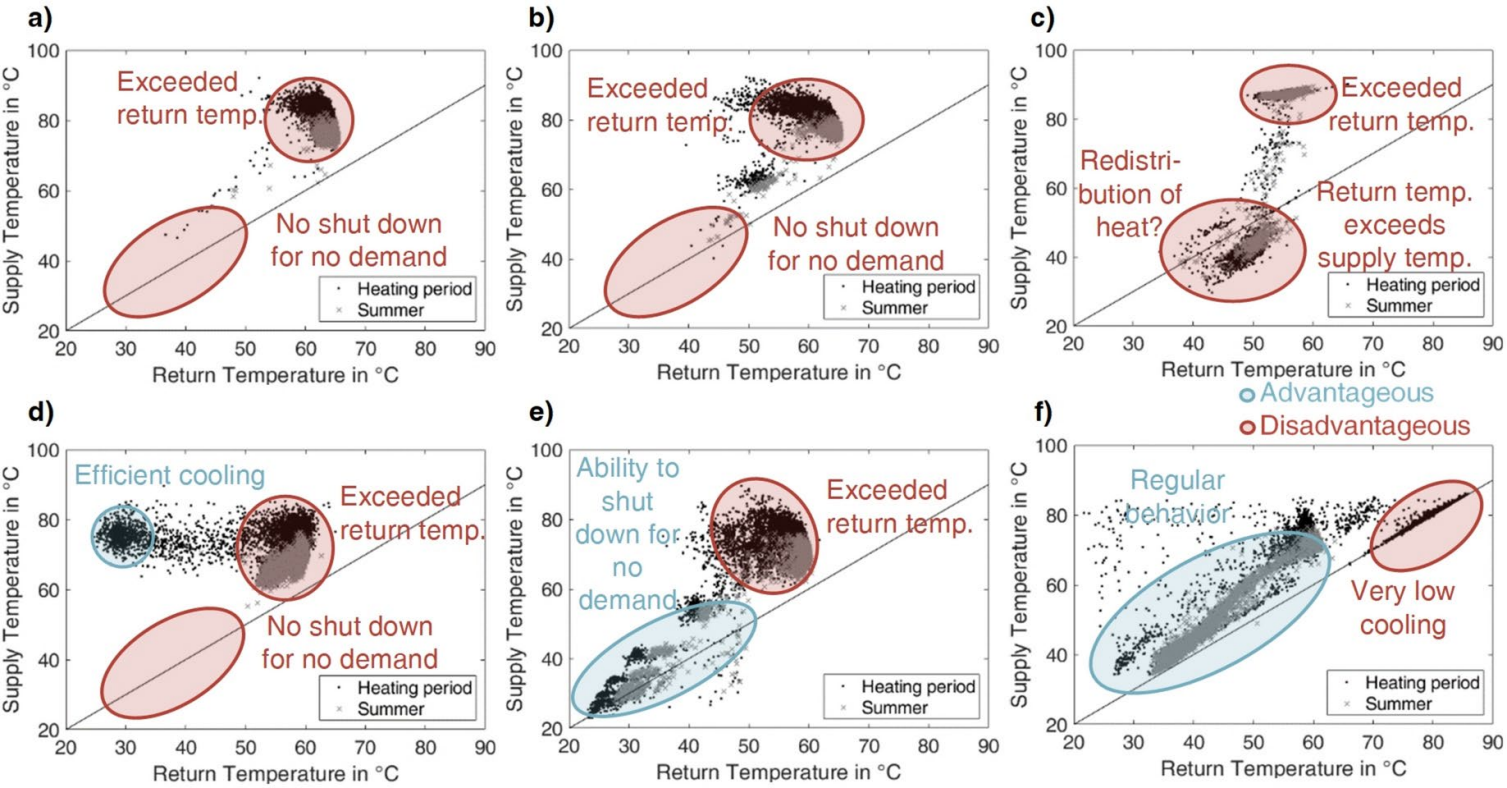
Selection of 6 temperature signatures of substations with strong deviation from general pattern. (**a**) to (**f**) distinguish between the heating period (black dots) and summer (grey crosses) with an indication of advantageous (blue) and disadvantageous (red) positions of data samples and a reference to the impact on the heating system, where the 6 selected substations originate from the 10 substations identified as most strongly deviating.

Figure 7 shows the temperature signatures of six of the ten substations, which are identified most often as providing unusual patterns (Fig. 6). The choice contains the most informative plots to cover all of the behavioral patterns indicative of faults identified as occurring most often.

The displayed datasets in Fig. 7 tend to exhibit accumulation in the upper right corner, which is disadvantageous for the reason of high return temperatures. As a legally binding return temperature limitation should strictly avoid more than 55 °C, each substation appears to show a fault leading to this behavior. Figure 7 a) and b) are neighboring substations of buildings. As Fig. 7 a) and b) appear to have similar temperature signatures, similar faults could have occurred. In the following, expert evaluation shall provide possible causes.

Figure 7 a) and b) exhibit very low cooling of the heat carrier. The control strategy or settings likely do not allow for a more advantageous operation.

For Fig. 7 c), many samples appear below the solid black line, indicative of recordings with a higher return than supply temperature. This behavior should occur only for a limited number of samples, which is equivalent to a low number of recorded hours for this state. Visits to the site revealed that a temperature signature like Fig. 7 c) indicates a faulty or at least disadvantageous control of the building’s heat storage, possibly redistributing heat into the system.

The substation in Fig. 7 d) evidently could reach sufficient cooling, but this state occurs comparatively rarely and only in the heating season. If the supply temperature level were low, the inefficient operation could be related to the location in the network, as discussed above. However, regarding Fig. 7 a), b), and d), the supply temperature is sufficient, especially in the heating period, and never falls below 60 °C, even in summer. Therefore, it must be concluded that the substations Fig. 7 a), b), and d) show a constant flow of the heat carrier, possibly because of direct heat supply for domestic hot water, but evidently also associated with low cooling when demand exists. Hence, the control strategy or the facilities’ architectures do not allow for higher cooling.

In contrast, Fig. 7 e) exhibits stagnation but appears unable to reach advantageous operating points when supplying heat, possibly for the same reasons as for Fig. 7 a), b), and d).

Figure 7 f) displays a particular case of behavior, hereby referred to as very low cooling. The volume flow is uncontrolled, and no heat is supplied for samples, indicating the same value for the supply and return temperatures (accumulation of data samples on the black line in the upper right corner). However, the fault leading to the very low cooling developed over time and during the heating period, which can be seen from the remaining data points. Yet, very low cooling should be distinguished from the behavior of a restarting facility, which can cause similarly low temperature differences but for lower temperature levels.

With expert knowledge, this study determined three prevailing types in the model region with the prevailing underlying causes in brackets:

1. Exceeded return temperature (disabled, modified, or non-existent return temperature limitation valve)
2. Very low cooling (defective or stuck flow control valve)
3. Inverted temperatures (defective or inadequate control of storage tank)

The very low cooling is a particular case of exceeded return temperatures, where the volume flow is not adjusted sufficiently but passes at a high rate in an uncontrolled or very limitedly controlled manner. Yet, as measures are often straightforward, a separate treatment of this behavior can be advantageous: As stated in Ref^7,17^., the motoric valves are prone to faults. Often, blocking causes a very low cooling. Ref^7^. names the first two indicators as well. However, the third case could be identified based on the clustering approach as an additional indicator.

### Statistical identification of faulty substations and quantification of their impact on the heat network

Based on the labeled patterns indicative of faults acquired by the clustering approach, statistically accessible features for the identification of faulty substations are generated:

1. The mean return temperature reflects whether this value surpasses the threshold of 55 °C specific to the heat network under investigation. The higher the volume flow is for these readings, the higher the impact on the system.
2. Similarly, very low cooling can be identified by evaluating the temperature difference between supply and return temperature readings. As a boundary condition, the very low cooling is generally characterized by a high temperature level. Again, the volume flow readings enable quantifying the impact on the system.
3. Thirdly, the inversion of the temperature potential can be evaluated. For this case, a return temperature measurement higher than the supply temperature is most disadvantageous for the heating system if it occurs for readings of the volume flow > 0, as heat is redistributed in this case.
  For an exceeded return temperature and very low cooling, this study quantifies the negative impact as follows. Firstly, only for a return temperature exceeding 55 °C on average does the operator have legal grounds to require measures. Employing this value as a reference is suggested. The mean value of the return temperature ${\overline{T} }_{2}$ is formed and compared to 55 °C. The higher this temperature difference, the stronger the negative impact of the fault on the system. Therefore, the theoretical value for the surplus of heat ${Q}_{55}$, which should have been transferred in the substation to reach 55 °C in the return flow, is calculated (Eq. 1).
  1 $${Q}_{55}=m\times {c}_{p}\times \left({\overline{T} }_{2}-55^\circ C\right)$$

$$m=\int_{t_{begin}}^{t_{end}}\dot{m}{(t)}dt$$

Calculating the inverse heat flow requires an additional step. Forming a mean value of the temperatures could lead to a leveling out of periods in which the potential has the expected direction ${(T}_{1}>{T}_{2})$ and periods of inversion $({T}_{2}>{T}_{1})$. If the temperature readings indicate an inversion, but if no primary volume flow occurs, no heat is transferred. Thus, the returned heat is calculated according to Eq. 2.

2 $${Q}_{ret}={\int }_{{t}_{begin}}^{{t}_{end}}{\dot{Q}}_{ret}\left(t\right) dt$$

with

$$\dot{Q}_{ret}(t)=\left\{\begin{array}{l} \dot{m}(t)\times c_p \times (T_2(t)-T_1(t))\quad \text{for} \quad T_2>T_1 \\ 0 \quad \text{for}\quad T_2 \leq T_1 \end{array} \right.$$

**Fig. 8:**
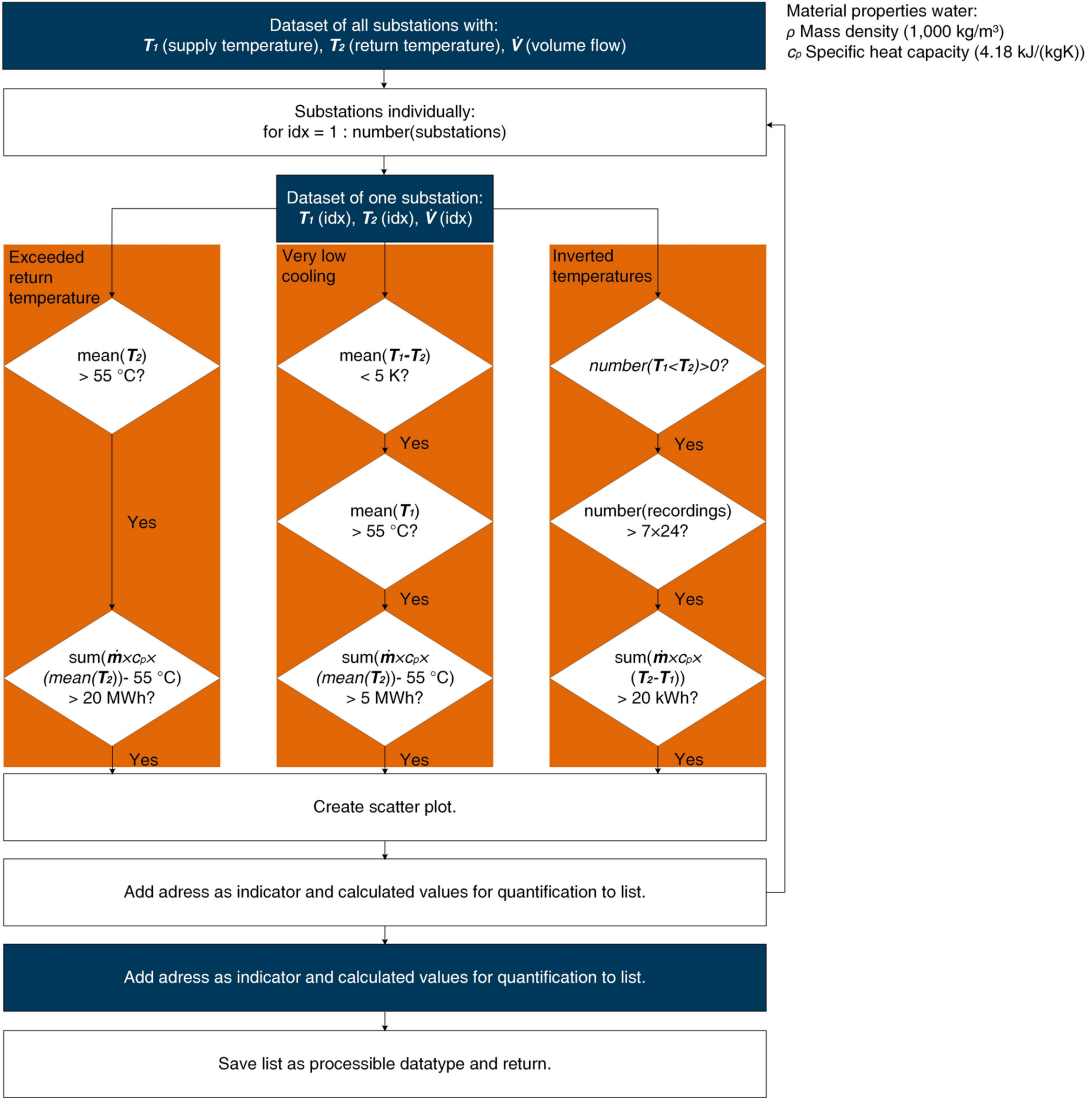
Algorithmic workflow of statistical approach for the identification and impact evaluation of faults on the user level. The data are processed individually for each substation in three paths to evaluate whether a fault exists and determine the degree of influence on the system with the named restrictions, after which the results are visualized and saved in a user-friendly manner.

Figure 8 outlines the explicit algorithmic flow chart for identifying faults and quantifying their impact resulting from the general framework in Fig. S4 (Supplementary Information). In the specific case of the model region, the integration is discretized in hourly timesteps, resulting in a summation replacing the integration.

Table 1 gives the results for executing the algorithm encoding steps 2 and 3 of the three-step method. The September 2023 data is employed exemplarily with the named thresholds and boundary conditions.

**Table 1:** Results of statistical fault detection and impact quantification exemplarily for data of September 2023.

Results: exceeded return temperature			Results: very low cooling		
Substation	Mean return temperature in °C	Surplus heat in return flow in MWh	Substation	Mean temperature difference in °C	Surplus heat in return flow in MWh
1	61.41	94.10	2	3.72	30.05
2	68.16	30.05	5	3.74	8.06
3	66.10	29.48	6	2.31	7.86
4	56.12	20.50	7	1.01	5.36

**Results: inverted temperatures and returned heat flow**					
Substation	Returned heat in kWh		Substation	Returned heat in kWh	
A	151.50		J	29.29	
B	99.99		K	28.42	
C	67.64		L	25.66	
D	48.06		M	25.62	
E	44.76		N	25.24	
F	42.13		O	23.73	
G	41.42		P	22.71	
H	36.33		Q	21.79	
I	30.97		R	20.81	

The assigned thresholds aim to maintain relevance regarding the operator’s limited fault management resources. Conducting fault detection without any threshold reveals a leveling out of the numerical value assigned to the respective impact of the faults. In the case of the explicit sample set, 20 MWh of heat exceeding 55 °C in the return flow for the first fault, 5 MWh for the very low cooling, and at least 20 kWh for the returned heat could be identified as suitable with manual analysis. Below the thresholds, a saturation within the values in Table 1 starts to occur.

The returned heat threshold is specifically lower in order of magnitude than for the other two faults. However, detecting malfunctions in the substation control can have a strong influence: As a fault in the substation control usually leads to inefficient behavior, the return temperature can exceed the limits for times of high demand. In contrast, heat is returned in times of low demand. This means that, on average, the threshold of 55 °C might not be exceeded, but the impact can be high nevertheless.

It should be mentioned that evaluating the heat flow for all three cases is expedient only if sufficient samples are collected. Otherwise, the results returned for the discrete summation are, evidently, lower. The summation of returned heat has shown to be especially sensitive to reading errors for low amounts of collected data. Thus, imposing a boundary condition in this case and exclusively considering substation datasets with, e.g., at least one week of readings is suggested.

The substations in the “very low cooling” column of Table 1 relate to the numbers for exceeded return temperature when sorted in descending order. Thus, the numbers indicate that the approach for the return temperature does indeed include very low cooling, but it does not necessarily appear amongst the highest priorities.

The substations that returned noticeably high amounts of heat did not appear in any of the other two lists of faulty substations in Table 1, which is why they are referred to alphabetically.

For the investigated period of September 2023, counting without the application of the threshold, a considerable share of 49 (10%) of the investigated 486 substations exhibit an exceeded return temperature. Yet, only a limited number of these seriously impact the system. Still, the necessity for measures is emphasized by the results.

### Special cases for temperature signatures related to faults and identified causes

Specific temperature signatures shall be addressed to outline the limits and chances of this study’s methods. The objective is to enhance expert knowledge and clarify information that can be derived from a substation’s temperature signature.

This study assigns causes to the visible temperature signatures in the context of labeling data. Subsequent research can aid in classifying faults automatically and reduce the necessity for manual processing even more. Also, the impact of the frequency of conducting fault detection becomes more apparent as the temperature signatures give indications of when the fault occurred.

**Fig. 9:**
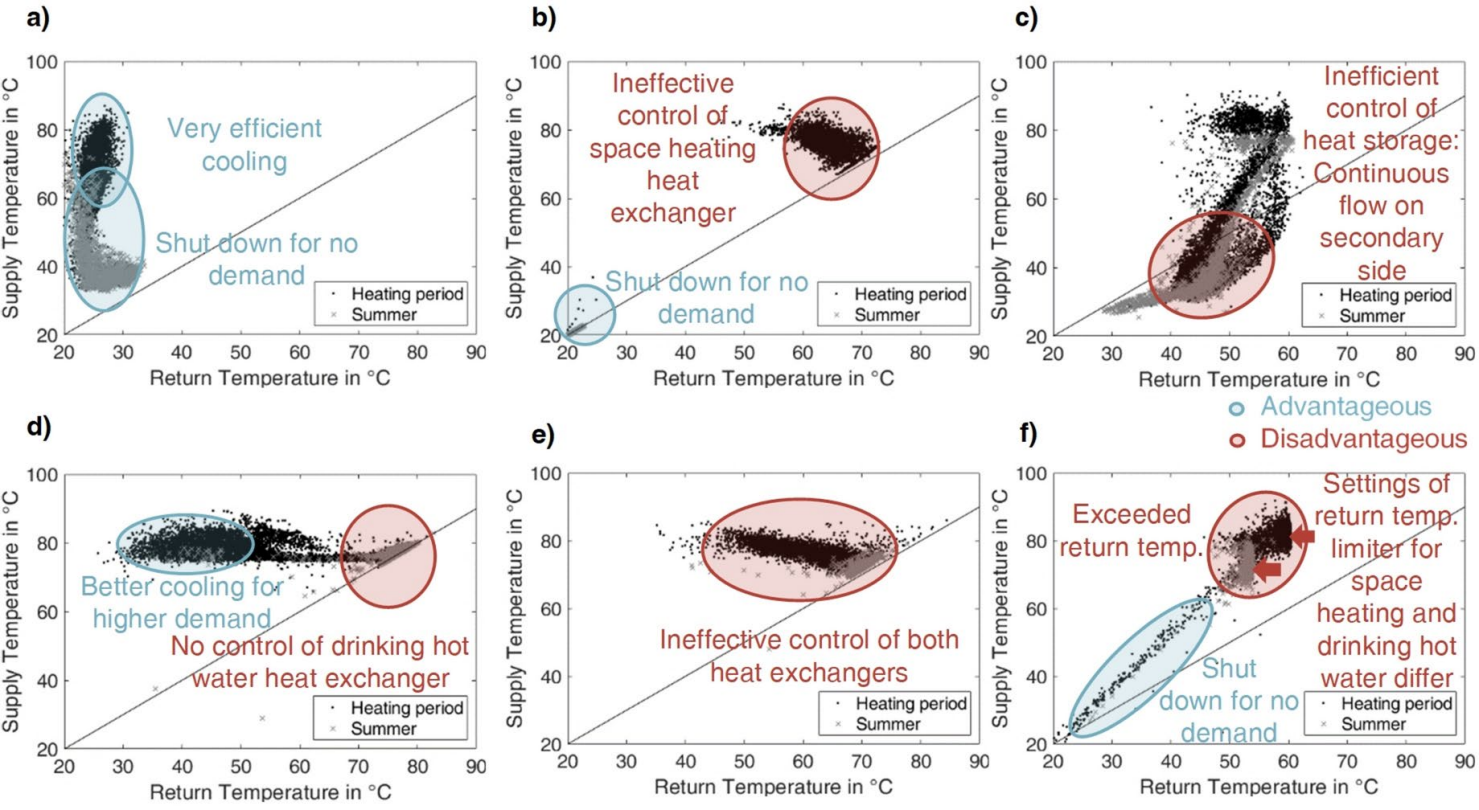
Special cases of end-user temperature signatures in the model region. (**a to f**) Distinguish between heating period (black dots) and summer (grey crosses) with an indication of advantageous (blue) and disadvantageous (red) positions of data samples and reference to the impact on the heating system.

Figure 9 a) to f) show temperature signatures of substations identified as showing a differing pattern or as indicative of a fault.

Figure 9 a) gives an example of the most advantageous operation in terms of system efficiency. Visibly, the end-user’s facility cools down very effectively, and the heat exchange stops during times of no demand, which occurs more often in summer. Naturally, the clustering approach identifies such a pattern as differing, but it is an anomaly with a positive effect on the system. Thus, for fault detection, the statistical method is superior in this case because it would correctly detect no fault.

In Fig. 9 b), the user evidently has no domestic hot water supply. A mere quantification of the temperature difference would lead to a false positive classification as a very low cooling case for the summer data. Thus, this study could provide the boundary condition of the general temperature level based on such patterns. Again, this emphasizes also the necessity of constant monitoring.

Figure 9 c) displays a case in which the return temperature frequently exceeds the supply temperature. A chain of circumstances leads to the pattern. The temperature control of the domestic hot water storage is disabled because the temperature sensor of the tank is not mounted correctly. As the pre-defined control program does not match, an alternative (unsuitable) program is selected. The return temperature limitation is very slow. By heat conduction, the resulting continuous volume flow of the hot water on the secondary side constantly warms the return temperature measuring probe, which is close to the heat exchanger in times of primary side stagnation. For system efficiency, this pattern is disadvantageous because of high return temperatures. User comfort is affected as well, as confirmed by the inhabitants. However, evaluating the average return temperature does not identify the fault, as the low return temperature values balance the very high ones. Thus, solely the evaluation of the temperature inversion this study established by using the clustering approach reveals the fault. The depicted temperature signature is referred to as R in Table 1. The investigation of the model region led to the conclusion that all substations on the road show the same chain of faults.

The patterns in Fig. 9 d) and e) firstly appear similar, yet the faults differ strongly. In Fig. 9 d), a visit revealed an uncontrolled heat exchanger for domestic hot water. An uncontrolled volume flow at its maximum constantly passes. However, the space heating facility shows no anomaly. Thus, the visible pattern is the sum of both. Especially in summer, the impact of the domestic hot water heat exchanger dominates.

In contrast, in Fig. 9 e), the control of both heat exchangers employs the return temperature limitation as an actor, and no other mechanism exists. The valve executing the limitation is very slow due to aging, so the control’s effect is considerably small. Thus, the threshold has been increased above 55 °C, assumingly to reestablish user comfort. Especially for times of no or low demand, the cooling of the heat carrier is very low, but the volume flow is not reduced to zero.

A comparable effect leads to the pattern in Fig. 9 f). The data samples show a boundary to the right for both summer and winter data. These are two different settings of the return temperature limitation valves in the heat exchangers for domestic hot water (the required 55 °C) and space heating (approximately 60 °C).

## Discussion

With the objective of maximizing automation and minimizing computational costs, this study first labeled the most frequently occurring behavioral patterns indicating faults in user substations with expert knowledge based on the clustering results. This enabled the development of features for a statistical fault detection process for the identified categories. The combination with the volume flow data provides a numerical value indicating the impact of the occurring fault on the system.

As shown above, the clustering approach aims to identify temperature signatures indicative of faults by assuming that faults lead to anomalous patterns in the recorded data. The clustering approach employs the temperature data only, as related research^6,15,20,23^, and relies on data accumulation instead of the dimensions of the volume flow and time. The principle does not depend on the explicit establishment of an expected behavior to distinguish between normal and anomalous behavior. Instead, the degree of deviation is determined by an evaluation of how often a specific pattern is identified as an anomaly by the clustering method. Encoding the employed data pre-preparation and clustering methods contributes to automation by reducing the manual processing steps and the number of substations to be manually investigated.

With the clustering approach, the features of a high return temperature and low cooling, known to indicate faults^7^, could be confirmed. A third feature, which has been focused on less so far, the inversion of the potential between supply and return temperature, could be identified. This was enabled by the clustering approach, which objectively returns the anomalous patterns regardless of prior knowledge. The third indicator proved beneficial in detecting additional faulty substations in the system. Hence, subsequent research could apply the principle to other model regions to provide additional features.

With statistically accessible labels for fault detection established, the suggested method for the detection of faulty substations works fully automated and with limited reliance on the sequential order of the time-series data. The faults’ impact on the heating network could be quantified automatically based on the volume flow readings. The method can potentially be used with limited adjustments for the detection process in other district heating networks. One requirement is sufficient user data recorded for the primary side of supply and return temperatures, as well as the volume flow in an adequate temporal resolution. The suggested threshold values in this study are case-specific. For example, the values of the calculated heat ${Q}_{55}$ and ${Q}_{\text{ret}}$depend on the considered period of one month and the dimensions of the heat network. The workflow is suitable for a network with data acquisition on the user level. However, if the measurements of the domestic hot water and the space heating facilities are separated, the process requires modifications, and different temperature signatures will occur. This study suggests executing the statistical fault detection process monthly, as applied in the literature^7^, to enable quick identification and avoid overlooking faults that occur only for certain demand situations.

The relevance of faults on the user’s secondary side has been showcased, e.g., in Ref^16,17^. In the model region, only the primary side user data is recorded. However, once the secondary side data become available on a broad scale, these can contribute additional labels for fault detection, providing another relevant area of interest.

Data quality and quantity inflict additional challenges. As displayed above, the model region cannot provide complete datasets of all substations. As stated, only 167 of the 486 provided a comparable amount of data of 90%. This underlines the necessity for high-quality data for the machine learning approaches again. Connections for wireless data transmission can be lost, especially in summer, as foliage interferes. The lost connections lead to more information collected on the heating season, and statements concerning the summer become more complex. Low rates of collected data can lead to misclassifications when detecting faults as statistical impacts grow in relevance.

Different reasons can explain similar patterns of temperature signatures. As shown by the results, the individuality of the facilities in historically grown heat networks poses the major challenge for identifying the cause leading to a specific temperature signature. Therefore, this article addresses the most common temperature signatures and assigns the most common underlying causes. However, expert knowledge should further contribute to providing underlying phenomena and labeling data.

Fault management contributes to a higher resolution of knowledge on thermal energy systems. The results of this study outline the direct benefit implied by successful fault detection for the energy efficiency of heat networks. Additionally, the indirect advantages of increasing data quality for modeling purposes to enhance system efficiency should be highlighted.

## Conclusions

This study’s suggested three-step workflow for fault management on the user level within heat networks aims to contribute to the automation of effective fault management methods using data from a model region in northern Germany. The workflow utilizes available primary-side system data (volume flow, supply, and return temperature) of substations on the user level. Clustering analysis first identifies fault-indicating data patterns, which are labeled with expert knowledge to provide statistically accessible features. Secondly, these features are used in an automated statistical detection process to identify defective substations. Thirdly, a numerical quantification of the fault’s impact on the heating system is calculated, relying on the thermodynamic relationship of the temperatures and the volume flow. 

The workflow clusters heat maps of the substations’ temperature signatures, which fuse the information of the volume flow and the time dependency with the temperature data. With expert knowledge, statistically accessible features for fault detection could be generated for common faults: an exceeded return temperature, very low cooling, and inverted temperatures.

Statistical analysis using the features determined with the clustering approach could automatically detect faulty substations within the model region. Disabled return temperature limitation units, defective motoric valves, and faults in the storage control form the most common underlying causes for faulty substation behavior. 

This study automatically quantifies the negative impact of a detected fault on the energy system regardless of the substation size by combining the temperature and volume flow data, which can enable the prioritization of fault elimination measures in practical applications. The workflow further outlines thresholds for maintaining relevance. 

This study shows that the temperature signature of substations is suitable for identifying data patterns indicating faults decisively impacting the efficiency of heat networks. The results validate that faulty user substations can be detected successfully with minimal manual processing. While the method proved effective, challenges remain in diagnosing faults remotely, emphasizing the need for further studies in diverse model regions to enhance data labeling.

Fault management increases user comfort and system efficiency and can have economic advantages while also improving data quality for machine and deep learning applications. Thus, the suggested method for fault detection contributes to enhancing fault management, improving data-driven modeling, and increasing operational efficiency in heat networks.

## Employment

Ulf Rieck-Blankenburg has been employed at the Stadtwerke Flensburg GmbH, while this study was conducted.

## Supplementary Information

Supplementary Information.

## Data Availability

Raw data are not publicly available for the model region dataset to preserve individuals’ privacy under the European General Data Protection Regulation. Anonymized datasets generated during the study are available from the corresponding author upon reasonable request and with the consent of the heating system operator.
