# Supplementary material for: Clustering- and statistic-based approach for detection and impact evaluation of faults in end-user substations of thermal energy systems
Source: Sci Rep. 2024 Dec 31;14:32166. doi: 10.1038/s41598-024-82103-5 (PMC11688455; doi:10.1038/s41598-024-82103-5)
Supplement: Supplementary file 1 — Supplementary Information. [file 41598_2024_82103_MOESM1_ESM.pdf]

Supplementary Information

Clustering- and statistic-based approach for detection and impact evaluation of faults in end-user substations of thermal energy systems

Samanta A. Weber [1,2]*, Michael Fischlschweiger [2], Dirk Volta [1] & Ulf Rieck-Blankenburg [3]

[1] Energy and Life Science, University of Applied Sciences Flensburg, 24943 Flensburg, Germany

[2] Chair of Technical Thermodynamics and Energy Efficient Material Treatment, Institute of Energy Process Engineering and Fuel Technology, Clausthal University of Technology, 38678 Clausthal-Zellerfeld, Germany

[3] Business Segment Networks, Stadtwerke Flensburg GmbH, 24939 Flensburg, Germany

*samanta.weber@hs-flensburg.de, samanta.weber@tu-clausthal.de, https://orcid.org/0000-0002-6487-8248

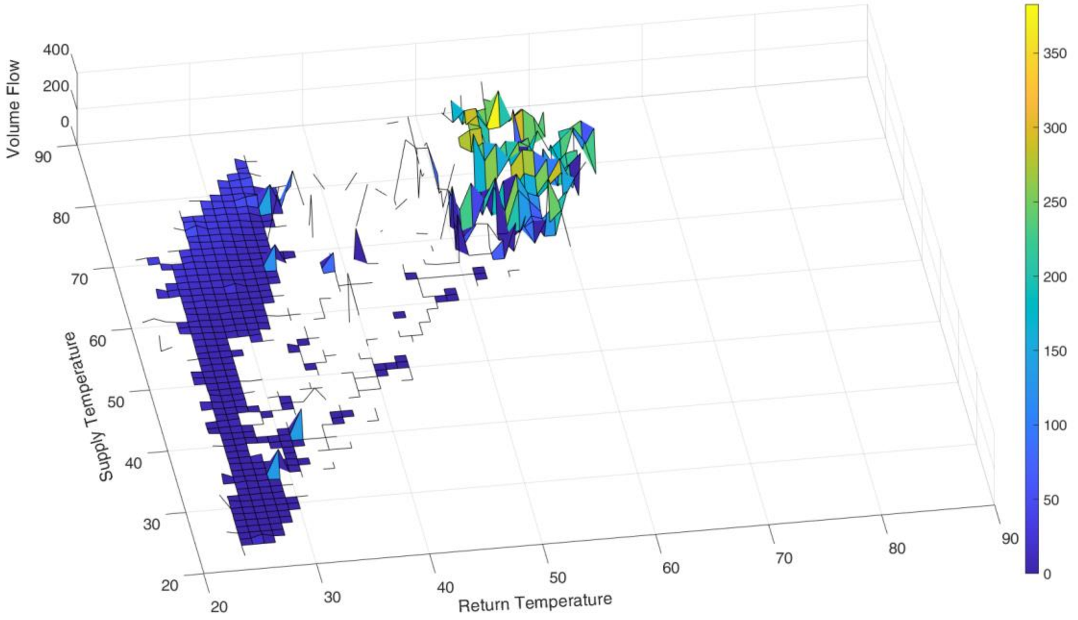

Figure S1 Volume flow data over supply and return temperature for data of substation in Fig. 4 a), b). High volume flow values occur for a high supply temperature associated with a high return temperature, as the high flow rate leads to reduced cooling of the heat carrier. Low volume flow values correlate with efficient cooling, which can be found on the left.

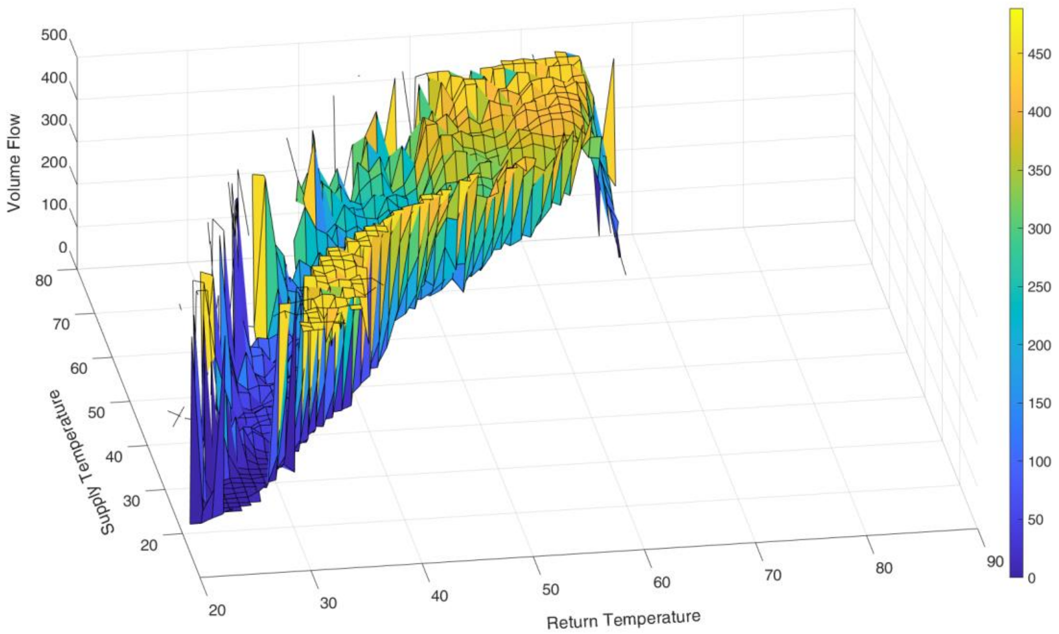

Figure S2 Volume flow data over supply and return temperature for data of substation in Fig. 4 c), d). The volume flow values are higher in general compared to Fig. S1. Consequently, the cooling is lower, and the data accumulates more on the right at higher values of the return temperature.

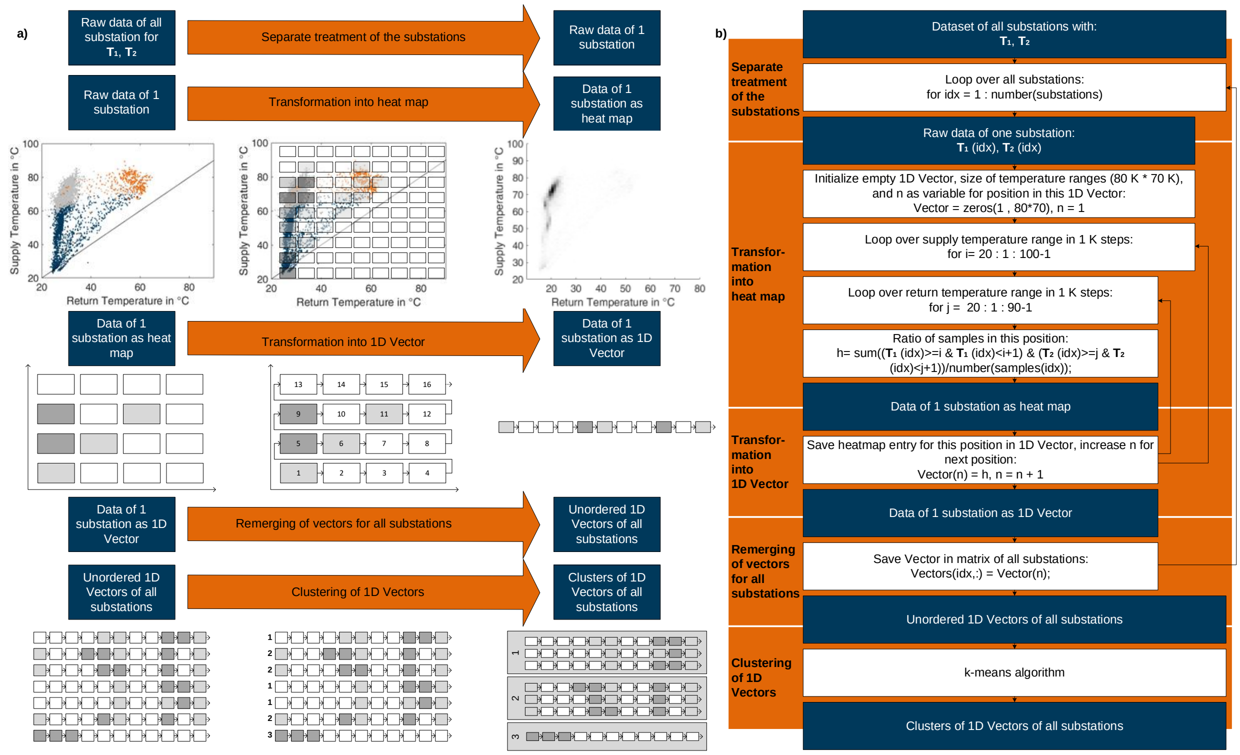

Figure S3 a) Visualization of processing steps for data pre-preparation and clustering with b) flow chart. The processing steps in orange and the data in dark blue in a) are mirrored by the flow chart in b).

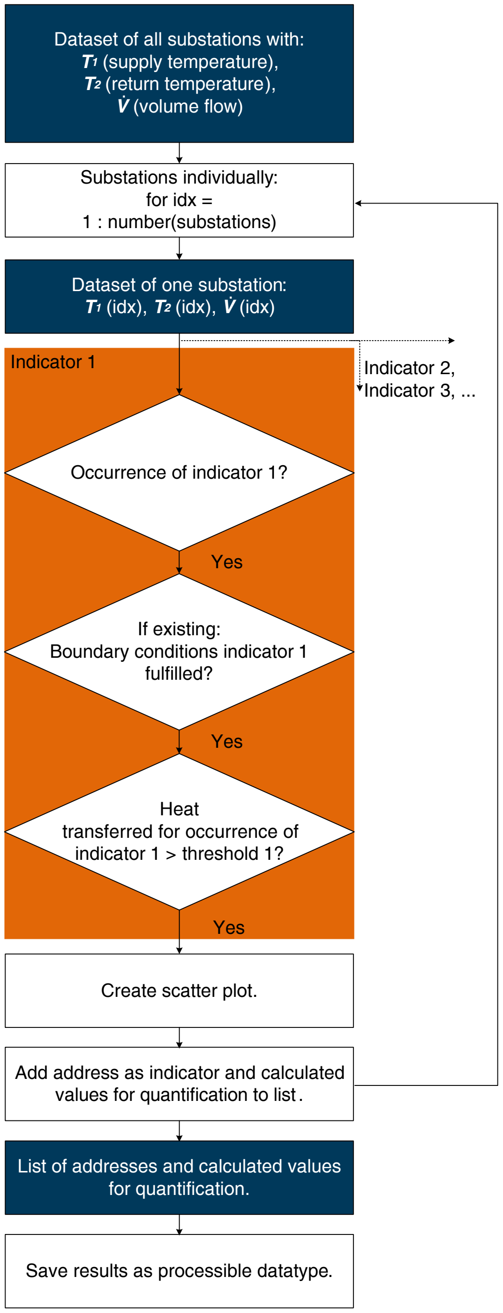

Figure S4 Flow chart for encoding of statistical fault detection and impact quantification. For each substation, each indicator for the occurrence of a fault is tested for fulfillment; if existing, indicator-specific boundary conditions are checked; the heat transferred for the occurrence of the indicator is calculated; if the amount is higher than the assigned threshold, the substation's address and computed values are returned.
